# Neighbor-Affected Orientation Rotation in the Grain Boundary Region

**DOI:** 10.3390/ma15031059

**Published:** 2022-01-29

**Authors:** Xi Chen, Yuhui Sha, Sihao Chen, Fang Zhang, Liang Zuo

**Affiliations:** Key Laboratory for Anisotropy and Texture of Materials (Ministry of Education), Northeastern University, Shenyang 110819, China; chenxineu@163.com (X.C.); chensihao1992@outlook.com (S.C.); zhangf@smm.neu.edu.cn (F.Z.); lzuo@mail.neu.edu.cn (L.Z.)

**Keywords:** orientation rotation, grain boundary, neighboring orientation, crystal plasticity, texture

## Abstract

Orientation rotation at grain boundary regions associated with neighboring orientations in Fe-3.0 wt.% Si non-oriented silicon steel has been investigated by crystal plastic simulation. Rotation tendency relative to a certain target orientation is evaluated by deviation angle variation. Taking ideal λ (<001>//ND, normal direction) as the target orientation, the deviation angle of scattered {001} <*uv*0> orientations at grain boundary regions affected by neighboring orientations during rolling is calculated and verified by experimental measurements. The rotation tendency and rotation velocity field at grain boundary regions are significantly changed by neighboring orientations. According to the neighbor affected orientation rotation, the initial texture can be precisely designed to control the deformation texture at grain boundary regions.

## 1. Introduction

Orientation rotation of grains during plastic deformation entails crystallographic texture development in polycrystals. Due to the interactions between grains, grain boundary regions usually exhibit a different orientation rotation from the grain interior [1,2,3]. Since grain boundary regions usually act as preferred recrystallization nucleation sites due to their advantages of stored energy and orientation gradient [4,5,6], the orientation rotation adjacent to grain boundary regions is crucial for texture control. Non-oriented silicon steel is an important magnetic material widely used in electrical equipment cores. The λ texture (<001>//ND, normal direction) is beneficial for magnetic properties of non-oriented silicon steel [7,8], but it is not a stable ending orientation in rolling [9,10,11] due to the divergent rotation of scattered λ orientations. Therefore, controlling the rotation of scattered λ orientations at grain boundary regions is always a challenge to optimize λ texture for non-oriented silicon steel.

The orientation rotation at grain boundary regions has attracted extensive attention. Raabe et al. [12,13] proposed that the initial orientations with high divergence are easily affected by neighboring grains. Tsuji et al. [14] found that {001}<110> oriented grains maintain a uniform orientation across grains even after serious rolling deformation, while stringer deformation bands appear at grain boundary regions of {001}<510>–<320> oriented grains. Similarity, Inagaki [15] observed in cold rolled iron alloy that {111}<*uvw*> oriented grains have an obvious orientation subdivision at grain boundary regions, while the neighboring {111}<110> oriented grains exhibit a consistent deformation orientation. Zaefferer et al. [16] utilized the misorientation angle between neighboring grains to represent the neighboring orientation effect and found that a strong orientation change occurs on both sides of the grain boundary with a large misorientation angle and no obvious orientation variation at grain boundary regions with a small misorientation angle. Mishra et al. [17] expressed the orientation rotation at grain boundary regions by Taylor factor difference between neighboring grains. In recent years, orientation rotation at grain boundary regions is well characterized by electron backscatter diffraction (EBSD). Subedi et al. [18] and Wright et al. [19] described the magnitude of orientation subdivision near a grain boundary by kernel average misorientation (KAM) and grain reference orientation deviation (GROD) based on EBSD. Signorelli et al. [20] and De Vincentis et al. [21] presented the width and sharpness of orientation gradient near a grain boundary by gradient average severity (GAS) and boundary effective thickness (BET). In addition, Nagarajan et al. [22] proposed that grain tends to rotate towards the neighboring orientation with the smallest misorientation angle, and this rotation tendency fails when the misorientation angle is greater than 30°.

The previous studies focused on describing the magnitude of orientation gradient and subdivision at a grain boundary region. However, the detailed rotation direction at a grain boundary region affected by neighboring orientation is usually neglected. From the view of texture control, deformation orientations at grain boundary regions are especially expected to converge to a preferred target orientation for better physical or mechanical properties [23]. The target orientation can be stable, metastable, or even unstable during deformation. Therefore, the rotation tendency relative to a target orientation at grain boundary regions becomes critically important.

Orientation rotation at grain boundary regions associated with neighboring orientations during rolling is investigated in the present study. Here, the ideal λ orientation in non-oriented silicon steel is chosen as the target orientation to evaluate the magnitude and direction of orientation rotation. Technologically, the present study aims to explore a method to optimize deformation texture at grain boundary regions.

## 2. Calculation Method

Orientation rotation relative to target orientation is evaluated by deviation angle variation. Before deformation, the deviation angle of any orientation *B* ($\varphi_{1}^{B}{,\Phi }^{B}{,\varphi }_{2}^{B}$) from a target orientation A ($\varphi_{1}^{A}{,\Phi }^{A}{,\varphi }_{2}^{A}$) can be calculated by misorientation matrix $M_{B\to A}$:

(1) $$\theta_{\mathrm{AB}}{=\mathrm{arcos}\{[\mathrm{tr}(M}_{B\to A})\;-\;1]/2\}$$

Given that orientation *B* rotates to orientation *B*’ ($\varphi_{1}^{B}+{\dot{\varphi }}_{1}^{B}\Delta t,\Phi^{B}+{\dot{\Phi }}^{B}\Delta t,\varphi_{2}^{B}+{\dot{\varphi }}_{2}^{B}\Delta t$) within strain Δε and time Δ*t*, where $ġ=({\dot{\varphi }}_{1},\dot{\Phi },{\dot{\varphi }}_{2})$ is the rotation vector. Then the deviation angle from target orientation after deformation can be calculated as:

(2) $$\theta_{\mathrm{AB}’}{=\mathrm{arcos}\{[\mathrm{tr}(M}_{B’\to A})\;-\;1]/2\}$$

and the deviation angle difference is:

(3) $$\Delta \theta =\theta_{\mathrm{AB}’}-\theta_{\mathrm{AB}}$$

Δθ > 0 indicates that initial orientation diverges from target orientation, while Δθ < 0 corresponds to a convergent rotation with respect to target orientation.

Orientation rotation during cold rolling is calculated by crystal plasticity. Deformation velocity gradient ***L*** can be decomposed into a symmetric plastic strain rate $\dot{\varepsilon }$ and an anti-symmetric material spin $\dot{W}$:

(4) $$L=\dot{\varepsilon }+\dot{W}$$

Plastic strain rate $\dot{\varepsilon }$ is contributed from slip rates $\dot{\gamma }$ of all slip systems:

(5) $$\dot{\varepsilon }=\frac{1}{2}\sum_{\alpha }^{K}(s^{\alpha }⊗m^{\alpha }+m^{\alpha }⊗s^{\alpha }){\dot{\gamma }}^{\alpha }$$

where unit vectors *s^α^* and *m^α^* are the slip direction and slip plane normal direction of *α*th slip system respectively, *K* is the total number of slip systems. The plastic spin caused by dislocation slip can be calculated as:

(6) $$\dot{\omega }=\frac{1}{2}\sum_{\alpha }^{K}(s^{\alpha }⊗m^{\alpha }-m^{\alpha }⊗s^{\alpha }){\dot{\gamma }}^{\alpha }$$

Then the lattice spin $\dot{\Omega }$ for orientation rotation is:

(7) $$\dot{\Omega }=\dot{W}-\dot{\omega }$$

Rotation vector $ġ=({\dot{\varphi }}_{1},\dot{\Phi },{\dot{\varphi }}_{2})$ can be calculated when lattice spin is defined with respect to sample reference frame [24]:

$${\dot{\varphi }}_{1}=-{\dot{\Omega }}_{12}-{\dot{\varphi }}_{2}\cos\Phi$$

(8) $$\dot{\Phi }=-{\dot{\Omega }}_{23}\cos\varphi_{1}-{\dot{\Omega }}_{31}\sin\varphi_{1}$$

$${\dot{\varphi }}_{2}=(-{\dot{\Omega }}_{23}\sin\varphi_{1}+{\dot{\Omega }}_{31}\cos\varphi_{1}/)\sin\Phi$$

For grain interior away from grain boundary, a rate-dependent model [25,26] is adopted to calculate slip rate:

(9) $${\dot{\gamma }}^{\alpha }={\dot{a}}^{\alpha }{\left|\frac{\tau^{\alpha }}{g^{\alpha }}\right|}^{n}\mathrm{sgn}(\tau^{\alpha })$$

where ${\dot{a}}^{\alpha }$ = 0.001 s^−1^ is reference strain rate and *n* = 20 is rate sensitivity exponent [27]. $\tau^{\alpha }$ and $g^{\alpha }$ are resolved shear stress and strength of *α*th slip system. Grain boundaries usually act as an obstacle to dislocation movement [28,29], so grain boundary obstacle stress (*τ*_obs_) is incorporated as resistance into slip system activation for grain boundary regions [30]:

(10) $${\dot{\gamma }}^{\alpha }={\dot{a}}^{\alpha }{\left|\frac{\tau_{\mathrm{eff}}^{\alpha }}{g^{\alpha }}\right|}^{n}\mathrm{sgn}(\tau_{\mathrm{eff}}^{\alpha })$$

(11) $$\tau_{\mathrm{eff}}^{\alpha }{=\tau }^{\alpha }-\tau_{\mathrm{obs}}^{\alpha }\;\;\;\;\;\;(\left|\tau^{\alpha }\right|{\;>\;\tau }_{\mathrm{obs}}^{\alpha })$$

(12) $${\;\tau }_{\mathrm{eff}}^{\alpha }=0\;\;\;\;\;(\left|\tau^{\alpha }\right|\leq \tau_{\mathrm{obs}}^{\alpha })$$

*τ*_obs_ can be calculated by slip transmissivity (*N*) at a grain boundary:

(13) $$\tau_{\mathrm{obs}}=(1\;-{\;N)\tau }^{*}$$

(14) $${N=(L}_{1}\cdot L_{i}{)\;\times \;(s}_{1}\cdot s_{i})$$

Slip transmissivity depends on grain boundary direction and slip systems geometry [31,32,33]. *L*_1_ represents the intersection line between grain boundary and slip plane of incoming dislocation. *L_i_* is the intersection line between grain boundary and slip plane of emitted dislocation in neighboring grain. *s*_1_ and *s_i_* are slip directions of incoming dislocation and emitted dislocation, respectively. The maximum obstacle stress of grain boundary $\tau^{*}$ is estimated to be 1.1 GPa [30]. Slip transmissivity ranges from 0 to 1, corresponding to the maximum and minimum obstacle stress. For a given incoming slip system, *τ*_obs_ is selected as the minimum value among all allowed emitted slip systems. Twenty-four slip systems (12 × {110}<111>, 12 × {112} <111>) are considered in body-centered cubic (bcc) non-oriented silicon steels.

Strain hardening is characterized by the increment of slip system strength $g^{\alpha }$ [34]:

(15) $$ġ=\sum_{\beta }h_{\alpha \beta }{\dot{\gamma }}^{\alpha }$$

(16) $${\;h}_{\alpha \alpha }=h\left(\gamma \right){=h}_{0}{\sec h}^{2}\left|h_{0}\gamma /{(\tau }_{s}-{\;\tau }_{0})\right|$$

(17) $$h_{\alpha \beta }=\mathrm{qh}(\gamma )$$

where $h_{\alpha \alpha }$ and $h_{\alpha \beta }$ (*α* ≠ *β*) are self and latent hardening modulus, respectively. $h_{0}$ = 60 is the initial hardening modulus, $\tau_{0}$ = 161 MPa and $\tau_{s}$ = 1137 MPa represent the yield stress and saturation stress of the slip system, respectively [27], *q* = 1.4 is a constant and *γ* is the cumulative shear strain on all slip systems.

## 3. Results

### 3.1. Comparison of Orientation Rotation between Calculation and Experiment

**Figure 1:**
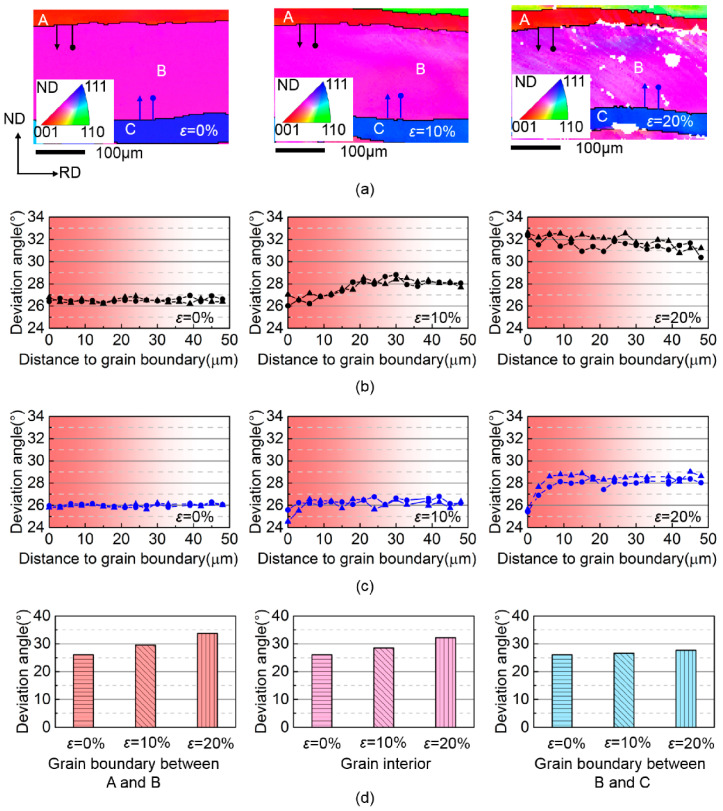
(**a**) Experimental orientation image maps of a selected zone in cold rolled Fe-3.0 wt.% Si non-oriented silicon steel sheet by EBSD, experimental deviation angle from ideal λ along the lines perpendicular to (**b**) grain boundary between grain A and B and (**c**) grain boundary between grain B and C, (**d**) calculated deviation angle from λ at grain interior and grain boundary regions of grain B. A B and C represent three grains, and *ε* denotes the cold rolling reduction.

A quasi in-situ EBSD analysis was conducted to trace the orientation rotation at grain interior and grain boundary regions in Fe-3.0 wt.% Si non-oriented silicon steel sheet, as shown in Figure 1.

The sample for EBSD was prepared by first mechanical polishing and then electropolishing in a solution of 96% ethanol and 4% perchloric acid for 20 s at 15 V to remove the deformed surface layer introduced by mechanical polishing. In quasi in-situ EBSD analysis, the initial orientations and position information of a selected zone were first recorded, and then the sample was cold-rolled using a rolling mill. After each 10% rolling reduction, the selected zone was measured again by EBSD without polishing. The grain orientations were observed by EBSD with a 3 μm step size on a JEOL JSM–7001F scanning electron microscope. The orientation image maps of the selected zone were analyzed by the HKL Channel 5 software. As shown in Figure 1a, initial grain A (*φ*_1_ = 75°, *Φ* = 10°, *φ*_2_ = 60°), grain B (*φ*_1_ = 47°, *Φ* = 26°, *φ*_2_= 40°) and grain C (*φ*_1_ = 38°, *Φ* = 47°, *φ*_2_ = 40°) are stacked with straight grain boundaries perpendicular to ND, so both grain interior and grain boundary regions can be assumed to experience the same rolling deformation velocity gradient in Equation (18). The deviation angle from ideal λ in grain B along the lines perpendicular to the grain boundary is quantified in Figure 1b,c according to the EBSD data. Then the corresponding deviation angles at grain interior and grain boundary regions of grain B are calculated in Figure 1d to compare with experimental measurements:

(18) $$L_{\mathrm{rolling}}=[\begin{array}{ccc} \dot{\varepsilon } & 0 & 0\\ 0 & 0 & 0\\ 0 & 0 & -\dot{\varepsilon } \end{array}]$$

The grain interior and grain boundary regions of grain B have nearly uniform 26° deviation angle from ideal λ prior to rolling. In both calculation and experiment, grain B has a divergent rotation relative to ideal λ at grain interior and grain boundary regions. After a 20% rolling reduction, the deviation angle affected by grain A is higher than grain interior while that affected by grain C is lower than grain interior, suggesting that the divergent rotation of grain B from ideal λ orientation is effectively decreased by neighboring grain C. The good agreement between experiment and calculation verifies the accuracy of the orientation rotation calculation model used in the present study.

### 3.2. Calculated Orientation Rotation at Grain Interior

**Figure 2:**
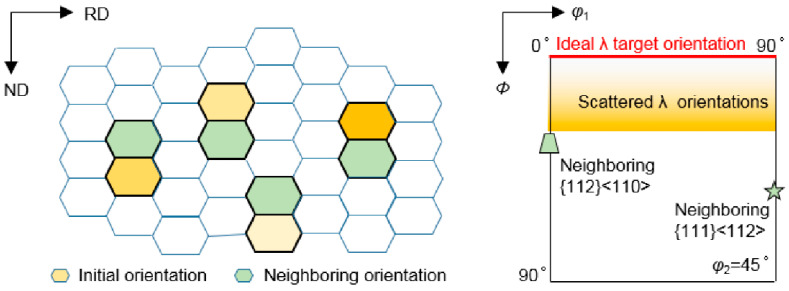
Schematic of grain stack for initial scattered {001} <*uv*0> orientations and neighboring orientations.

In order to compare the orientation rotation of various initial orientations at grain interior and grain boundary regions affected by neighboring orientations, the initial scattered {001} <*uv*0> orientations cover *φ*_1_ = 0°~90°, *Φ* = 0~30° and *φ*_2_ = 45° in Euler space, which deviate from ideal λ target orientation (*φ*_1_ = 0°~90°, *Φ* = 0°, *φ*_2_ = 45°) with *Φ* = 0°~30°. And typical {112}<110> (*φ*_1_ = 0°, *Φ* = 35°, *φ*_2_ = 45°) and {111}<112> (*φ*_1_ = 90°, *Φ* = 55°, *φ*_2_ = 45°) orientations with large volume fractions in rolled bcc metal are selected as the neighboring orientations. As seen in Figure 2, a pair of initial orientation and neighboring orientation is simplified to be stacked along ND in the calculation.

**Figure 3:**
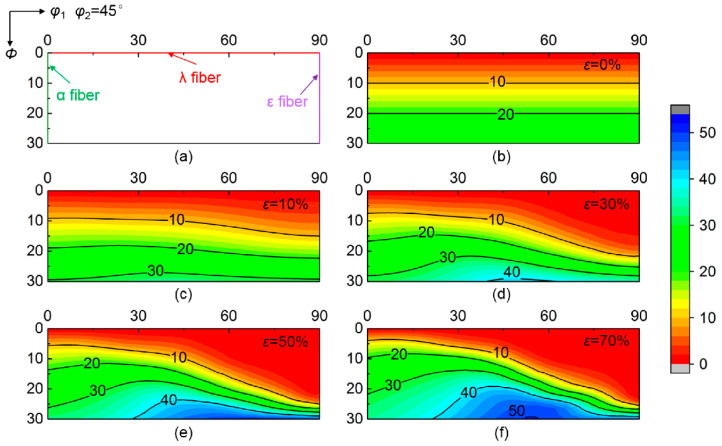
(**a**) Main orientation positions on *φ*_2_ = 45° section and (**b**–**f**) deviation angle from ideal λ target orientation of scattered {001} <*uv*0> orientations at grain interior during rolling.

**Figure 4:**
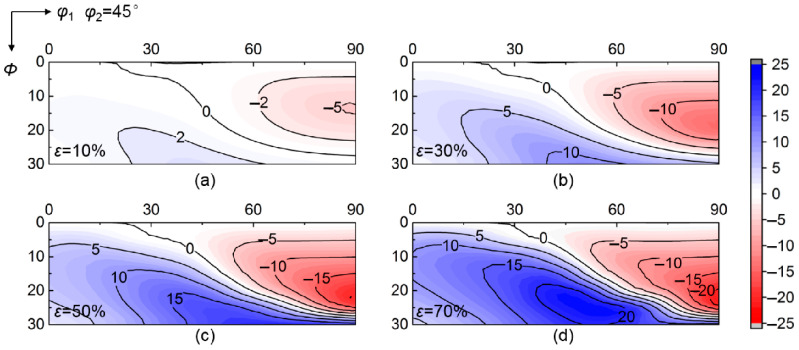
Deviation angle difference between *ε* = 0% and (**a**) 10%, (**b**) 30%, (**c**) 50%, (**d**) 70% rolling reductions at grain interior.

Figure 3 shows the calculated deviation angle from target orientation of various initial orientations at grain interiors during rolling up to 70% reduction. Various convergent and divergent rotations relative to target orientation occur at grain interiors during rolling. The deviation angle of initial orientations near ε fiber (<110>//TD, transverse direction) gradually decreases as strain increases, indicating the convergence to ideal λ target orientation. In contrast, the deviation angle of initial orientations around α fiber (<110>//RD, rolling direction) continuously increases during rolling, meaning the divergence from ideal λ target orientation. Figure 4 further gives the corresponding deviation angle difference Δ*θ* between various rolling reductions and *ε* = 0%. The magnitude of Δ*θ* in the convergent zone (Δ*θ* < 0) or divergent zone (Δ*θ* > 0) varies with initial orientations. In addition, the critical orientation boundary Δ*θ* = 0 separating convergent zone and divergent zone keeps nearly stable during rolling.

### 3.3. Calculated Orientation Rotation at Grain Boundary Region

**Figure 5:**
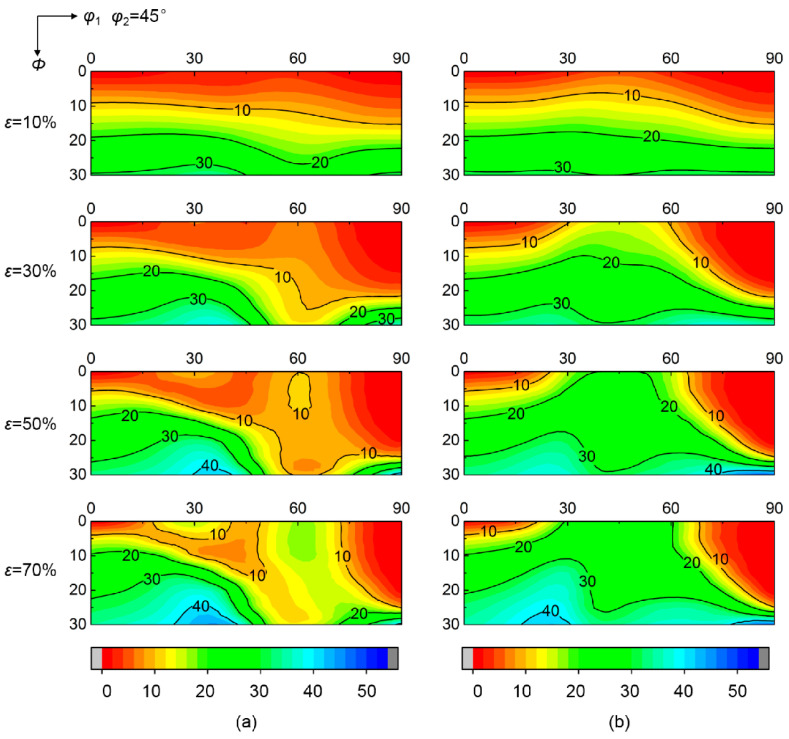
Deviation angle of various initial orientations from ideal λ target orientation at grain boundary regions affected by (**a**) {112}<110> and (**b**) {111}<112> neighboring orientations during rolling.

Figure 5 shows the calculated deviation angle of initial scattered {001} <*uv*0> from ideal λ target orientation at grain boundary regions affected by {112}<110> and {111}<112> neighboring orientations. Compared to grain interiors, the orientation rotation at grain boundary regions is obviously changed by neighboring orientations. For initial orientations within *φ*_1_ = 40°~60° and *Φ* = 0°~20°, the deviation angle at grain interiors decreases with strain, while the deviation angle at grain boundary regions gradually increases when adjacent to {111}<112> orientation, indicating that the rotation tendency is transformed from convergence at grain interiors into divergence at grain boundary regions. Conversely, the initial orientations with *φ*_1_ = 50°~70° and *Φ* = 20°~30° exhibit a change from the divergent rotation at grain interior into a convergent rotation at grain boundary regions by {112} <110> neighboring orientation. Furthermore, although the initial orientations with *φ*_1_ = 60°~75° still present a convergent rotation at grain boundary regions adjacent to {112} <110> orientation, the deviation angle is slightly increased compared with grain interiors.

**Figure 6:**
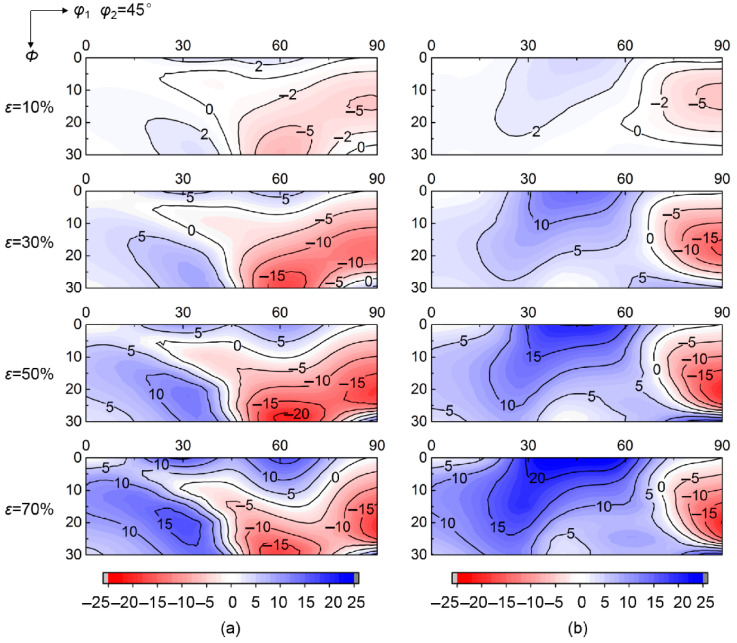
Deviation angle difference between *ε* = 0% and various rolling reductions at grain boundary regions affected by (**a**) {112}<110> and (**b**) {111}<112> neighboring orientations.

Figure 6 shows the deviation angle difference at grain boundary regions after various rolling reductions. Both the deviation angle and critical orientation boundary at grain boundary regions have a high sensitivity to neighboring orientation. The {112}<110> neighboring orientation produces an extended convergent zone and a shrunk divergent zone compared with {111}<112> neighboring orientation. Therefore, the rotation tendency relative to target orientation at grain boundary regions can be significantly modified by neighboring orientation.

## 4. Discussion

### 4.1. Orientation Rotation at Grain Boundary Region

Based on Figure 4 and Figure 6, significant changes take place in peak number and position of deviation angle difference before and after rolling, although the peak amplitude, ~20° at 50~70% reduction, is not affected obviously by neighboring orientation. By {112}<110> neighboring orientation, the divergent peak at *φ*_1_ = ~60° and *Φ* = ~30° in grain interior is reversed to convergent peak at grain boundary region, and there appear three new divergent peaks located at *φ*_1_ = ~30° and *Φ* = ~20°, *φ*_1_ = ~30° and *Φ* = ~0°, *φ*_1_ = ~60° and *Φ* = ~0°, respectively. In the case of {111}<112> neighboring orientation, the divergent peak moves from *φ*_1_ = ~60° and *Φ* = ~30° to *φ*_1_ = ~45° and *Φ* = ~0°. In addition, the convergent peak at *φ*_1_ = ~90° and *Φ* = ~20° is not affected by both {112}<110> and {111}<112> neighboring orientations.

**Figure 7:**
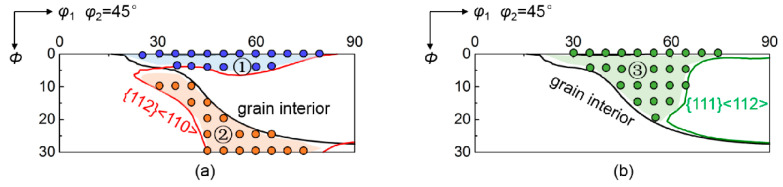
Shift of critical orientation boundary under the effect of (**a**) {112}<110> and (**b**) {111}<112> neighboring orientations. ①, ② and ③ denote the initial orientation zones affected by neighboring orientations.

Figure 7 shows the shift of the critical orientation boundary (Δ*θ* = 0) by neighboring orientations. The critical orientation boundary remains nearly stable at grain interior during rolling, while it is sensitive to neighboring orientations at grain boundary regions. In the case of {112}<110> neighboring orientation, the critical orientation boundary moves towards lower *φ*_1_ so that the orientation zone ② turns from divergence at grain interiors to convergence at grain boundary regions. While the shift of critical orientation boundary moves towards higher *Φ* changes the orientation zone ① from convergence at grain interior to divergence at grain boundary region. In contrast, the critical orientation boundary moves towards higher *φ*_1_ by {111}<112> neighboring orientation, transforming the orientation zone ③ from convergence at grain interior to divergence at grain boundary region.

### 4.2. Rotation Velocity Field at Grain Boundary Region

**Figure 8:**
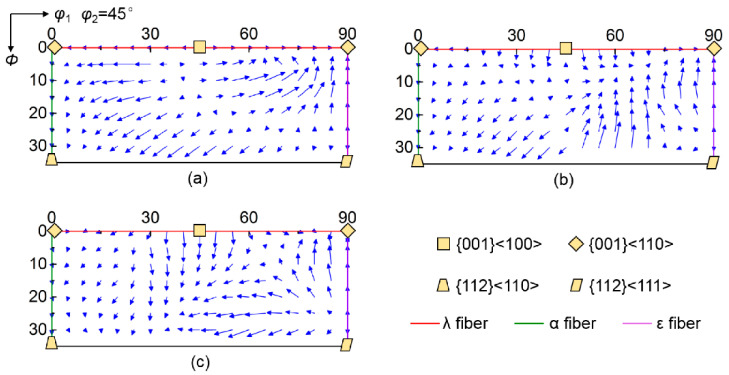
Rotation velocity field of various initial orientations at (**a**) grain interior and grain boundary regions affected by (**b**) {112}<110> and (**c**) {111}<112> neighboring orientations. Arrows denote the magnitude and direction of the rotation vector.

The special rotation tendency relative to target orientation at grain boundary region is actually attributed to rotation velocity field different from grain interior, which represents the rotation path and rate in Euler space. Figure 8 shows the rotation velocity field of scattered {001}<*uv*0> orientations at grain interior and grain boundary regions, where arrows denote the magnitude and direction of the rotation vector. At grain interior, initial orientations near ɛ fiber rotate towards {001}<110> orientation, so they present a decreasing deviation angle from λ fiber during rolling. Conversely, initial orientations near α fiber rotate towards {112}<110> orientation, leading to an increasing deviation angle from λ fiber. The rotation velocity field at grain boundary regions is sensitive to neighboring orientation. At grain boundary region adjacent to {112}<110> orientation, initial orientations near {001}<100> rotate away from λ fiber, while initial orientations within *φ*_1_ = 50°~70° and *Φ* = 20°~30° rotate towards lower *Φ*. For the grain boundary region adjacent to {111}<112> orientation, initial orientations around {001}<100> have a strong tendency to rotate towards higher *Φ*.

### 4.3. Correlation between Deviation Angle and Rotation Path

**Figure 9:**
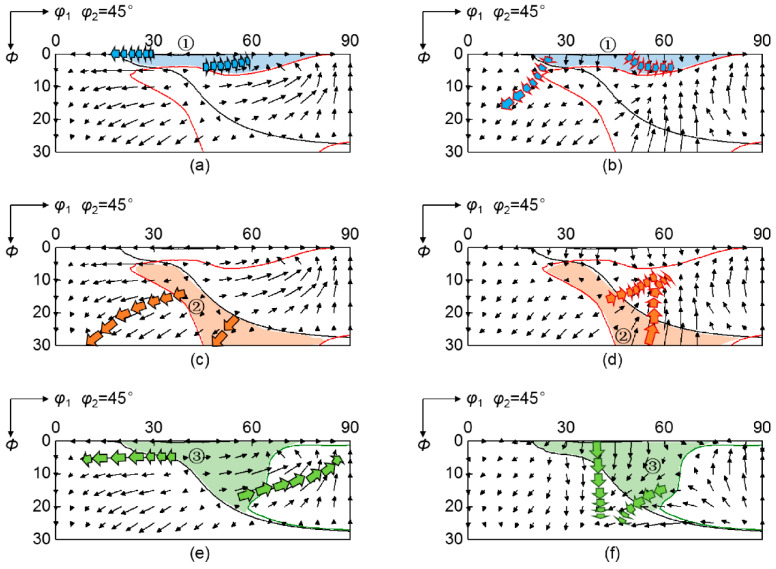
Rotation path of ①, ② and ③ orientation zones in Figure 7 during rolling at (**a**,**c**,**e**) grain interior and grain boundary regions affected by (**b**,**d**) {112}<110> and (**f**) {111}<112> neighboring orientations respectively. Arrows denote the magnitude and direction of the rotation vector.

During deformation, initial orientations flow in Euler space and pass through orientation zones with various rotation rates relative to the target orientation, so the variation of deviation angle with rolling reduction depends on the rotation path. Figure 9 shows the rotation path of zones ①, ② and ③ in Figure 7 at grain interior and grain boundary regions. Since ideal λ fiber covers *Φ* = 0° line, the decomposition of rotation vectors along *Φ* direction can roughly characterize the rotation rates relative to ideal λ. Initial orientations in zone ① rotate away from ideal λ fiber at grain boundary region affected by the {112}<110> neighboring orientation, and the divergent rotation rate increases during rolling. While initial orientations in zone ② converge to ideal λ with a decreasing rotation rate with rolling reduction by {112}<110> neighboring orientation. Initial orientations in zone ③ rotate away from ideal λ at grain boundary region affected by {111}<112> neighboring orientation, and the divergent rotation rate continually decreases during rolling. Therefore, appropriate initial orientations converge to λ target fiber under the influence of neighboring orientations, which can optimize the deformation texture at grain boundary regions in non-oriented silicon steels.

The neighbor-affected rotation velocity field moves the critical orientation boundary by changing the range of convergent and divergent orientation zones. Furthermore, the dramatic transformations in rotation rate and path at grain boundary regions create new peaks of deviation angle relative to target orientation, which vary with neighboring orientation relationship. Therefore, the quantitative characterization of rotation velocity field and deviation angle variation is valuable in designing initial texture to efficiently control orientation evolution at grain boundary regions during deformation.

## 5. Conclusions

(1) The rotation velocity field at grain boundary regions is quantitatively described by crystal plasticity calculations. Both the rotation path and rotation rate at grain boundary regions depend sensitively on initial orientation and neighboring orientation.
(2) Deviation angle evolution of initial scattered λ texture relative to ideal λ target orientation at grain boundary regions is sensitive to neighboring orientations. The critical orientation boundary separating convergent and divergent zones and the peak position of orientation zones can be effectively modified by neighboring orientations.
(3) Rotation velocity field and deviation angle distribution dependent on neighboring orientation provide a basis for accurate texture design to control orientation evolution at grain boundary regions during deformation.

## Data Availability

The raw/processed data can be available from the corresponding author on a reasonable request.
